# Identification of the key genes and immune infiltrating cells determined by sex differences in ischaemic stroke through co‐expression network module

**DOI:** 10.1049/syb2.12039

**Published:** 2021-11-18

**Authors:** Haipeng Xu, Yanzhi Ge, Yang Liu, Yang Zheng, Rong Hu, Conglin Ren, Qianqian Liu

**Affiliations:** ^1^ The Third Clinical Medical College Zhejiang Chinese Medical University Hangzhou Zhejiang China; ^2^ The First Affiliated Hospital Zhejiang Chinese Medical University Hangzhou Zhejiang China; ^3^ The Second Clinical Medical College Heilongjiang University of Chinese Medicine Harbin Heilongjiang China; ^4^ Department of Respiratory The First People's Hospital of Lanzhou City Lanzhou Gansu China

**Keywords:** differently expressed genes, ischaemic stroke, sex differences, WGCNA

## Abstract

Stroke is one of the leading causes of patients' death and long‐term disability worldwide, and ischaemic stroke (IS) accounts for nearly 80% of all strokes. Differential genes and weighted gene co‐expression network analysis (WGCNA) in male and female patients with IS were compared. The authors used cell type identification by estimating relative subsets of RNA transcripts (CIBERSORT) to analyse the distribution pattern of immune subtypes between male and female patients. In this study, 141 up‐regulated and 61 down‐regulated genes were gathered and distributed into five modules in response to their correlation degree to clinical traits. The criterion for Gene Ontology (GO) term and Kyoto Encyclopaedia of Genes and Genomes (KEGG) pathway indicated that detailed analysis had the potential to enhance clinical prediction and to identify the gender‐related mechanism. After that, the expression levels of hub genes were measured via the quantitative real‐time PCR (qRT‐PCR) method. Finally, CCL20, ICAM1 and PTGS2 were identified and these may be some promising targets for sex differences in IS. Besides, the hub genes were further verified by rat experiments. Furthermore, these CIBERSORT results showed that T cells CD8 and Monocytes may be the target for the treatment of male and female patients, respectively.

## 1 INTRODUCTION

Stroke, a kind of nervous system disease, is caused by blood supply failure to the brain or blood vessel rupture in the brain [1]. According to the relevant reports, about 6 million people die from stroke every year (more than 10% of all deaths). Ischaemic stroke (IS), caused by artery occlusion, was the main type of strokes (about 87% of all stroke cases) and nearly half of deaths were caused by it [2]. More worryingly, two‐thirds of stroke survivors still had serious sequelae that imposed a heavy burden on society and families [3]. At present, tissue plasminogen activator (tPA), surgical and mechanical thrombectomy are important treatment methods for IS [4]. However, the above methods have a series of problems such as short optimal treatment time, high recurrence rate and limited recovery of neural function [5]. Therefore, there is an urgent need for a new treatment that can better promote neurological recovery and reduce the incidence of disease, but the molecular mechanism of it remains unclear. As some researchers showed, IS patients have significant sex differences, which can affect their clinical manifestations, epidemiologic features, pathophysiological characteristics, prognosis and results [6]. A new study also found that there was a complex interaction between nerve inflammation and the immune system. Immunotherapy, as a new type of treatment, can slow the disease progression, improve nerve function and prognosis [7], which deserved further study. But systematic studies on sex differences in stroke patients and distribution patterns of immune cell sub‐types remain in the exploration stage.

With the wide application and continuous development of gene chip technology, weighted gene co‐expression network analysis (WGCNA) has been extensively used to analyse a large amount of gene expression data. It can transform gene expression data into co‐expression modules, which plays a great role in comparing differently expressed genes (DEGs) and exploring the interaction on co‐expression module genes [8]. Cell type identification by estimating relative subsets of RNA transcripts (CIBERSORT) is a deconvolution algorithm, which can analyse each immune cell sub‐type and accurately quantify different immune cell components [9]. In this study, differential genes in male and female patients with IS were compared, and then co‐expression networks for sex differences were constructed through WGCNA. We tried to identify the key genes that distinguish male and female patients from the highly correlated cluster of genes in the module. Next, a total of 10 hub genes were identified with protein‐protein interaction (PPI) network. To verify this hypothesis, rats were subjected to middle cerebral artery occlusion (MCAO) to produce an ischaemic stroke model. MCAO is a well‐established model of focal ischaemic stroke in rodents, which is widely used in basic research of cerebral ischaemia diseases [10]. We validated the expression of 10 hub genes with a quantitative real‐time PCR (qRT‐PCR) to confirm the accuracy of our analysis. Moreover, we used CIBERSORT to further investigate the distribution pattern of peripheral blood immune cell subtypes in males and females with IS. In conclusion, our study helps to reveal the sex differences in IS, and lays a foundation for exploring the immune‐related signalling pathway mechanism of the disease and formulating the immunomodulatory therapy for it.

## 2 MATERIALS & METHODS

### 2.1 Data set information

Gene expression data was downloaded from the Gene Expression Omnibus (GEO) database (https://www.ncbi.nlm.nih.gov/geo/), with data set GSE22255 based on GPL570, 10 cases from IS male serum samples, 10 from IS female serum samples and 20 from the health control. According to the task of this study, 10 male and 10 female patients with IS were selected for further analysis [11]. In addition, both GSE16561 and GSE37587 were downloaded to perform gender‐specific immune infiltration analysis on IS patients [12, 13]. The details of these microarray data were listed in Table 1. First, the expression matrix files of the two data sets downloaded from the GEO database were normalised and log2 transformed. Next, we matched the platform annotation files with each probe expression matrix, and finally, the ‘sva’ package was used to eliminate the differences between all batches of the included samples, thus ensuring the accuracy of the data.

**TABLE 1:** Identification of the key genes and immune infiltrating cells of sex difference in IS

GEO database	Platform	Samples	Number of cases	Number of controls
GSE22255	GPL570	PBMCs	20 IS samples	20 healthy controls
GSE16561	GPL6883	Whole blood samples	39 IS samples	24 healthy controls
GSE37587	GPL6883	Whole blood samples	68 IS samples	NS

Abbreviations: NS, not stated; PBMCs, peripheral blood mononuclear cells.

### 2.2 Identification of DEGs in male and female patients with IS

R software and related R package were used to normalise and analyse DEGs. The screening criteria for DEGs were as follows: log2 fold change (log2FC) should be greater than 2 or less than 2 and adjusted *p*‐value <0.05. The analysis results were presented by heat map and volcano map drawn in R Studio software (version: 1.2.1335). Subsequently, the most clinically significant modules and differential genes were selected for Venn diagram drawing and the intersection genes for subsequent analysis.

### 2.3 Construction of co‐expression networks

The ‘WGCNA’ programme package in R language was used to identify the co‐expressed gene modules, divide the samples into modules through the heat map toolkit analysis, and visualise all modules [14]. First, samples were clustered to evaluate whether there were any obvious outliers. Second, automatic network construction was used to construct the co‐expression network. Third, hierarchical clustering and dynamic tree‐cutting function detection module were used. Fourth, Gene Salience (GS) and Module Membership (MM) were calculated to correlate modules with clinical characteristics. Finally, the most clinically significant gene information was selected for further analysis.

### 2.4 Gene Ontology (GO) and Kyoto Encyclopaedia of Genes and Genomes (KEGG) analysis

Differential genes of the intersection were selected and genes of this part were dealt with enrichment analysis, to explore the functions of each module. The criterion for GO term and KEGG pathway was *p* threshold value <0.05.

### 2.5 Immune infiltration analysis with CIBERSORT

To explore the difference in the proportion of immune cell infiltration in male and female patients with IS, GSE16561 and GSE37587 were analysed using the CIBERSORT algorithm, and a total of 22 kinds of infiltrating immune cells were identified.

### 2.6 Construction of PPI network and identification of candidate hub genes

Based on the results above, the intersection genes were imported into the STRING website for further analysis. The lowest interaction score should be greater than 0.4 and isolated nodes in the network were removed. Then, the analysis results were output to the TSV format file and the details processing and module analysis were carried out by Cytoscape software (version: 3.7.1). Cytohubba is a plug‐in downloaded from the Cytoscape application store, which can find closely connected nodes in a complex network based on topology. Therefore, Cytohubba was used to detect the 10 most core genes in the PPI network.

### 2.7 Experimental animals and groups

In this study, 3–4 month healthy male and female SD rats were selected, with 10 rats in each gender and weighed 200 ± 50 g, from Shanghai Xipu Bikai experimental animal company (animal license No:SCXK (Shanghai) 2018‐0006), which in line with the standards of laboratory animals issued by the Ministry of Health of the People's Republic of China. The animal feeding, operation and sampling involved in this study all complied with the relevant regulations on the management and protection of experimental animals, which conformed to the ethical standards for animal experiments.

### 2.8 Model preparation method

**FIGURE 1:**
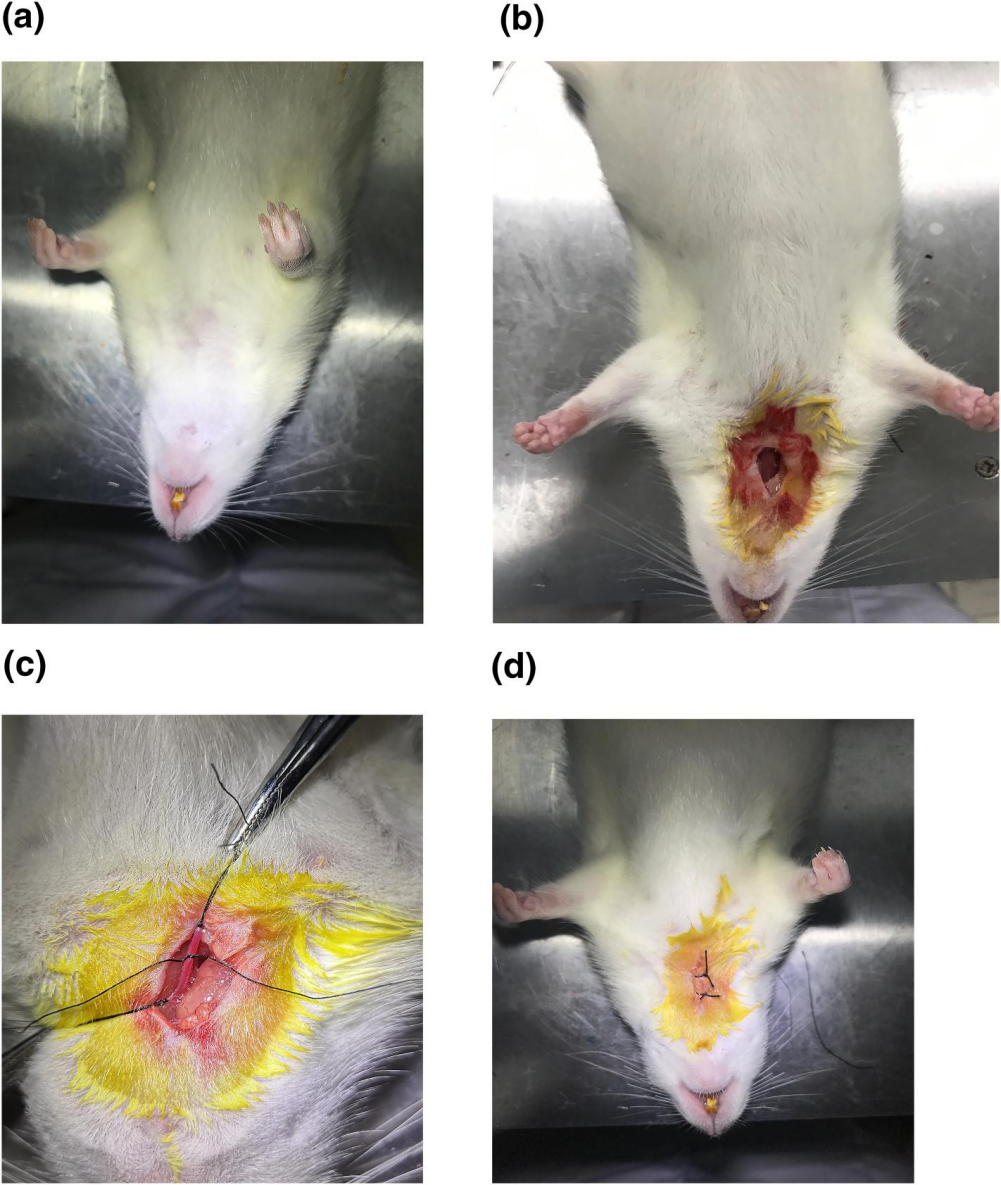
(a) The surgical site was exposed. (b) The operative area was disinfected and the tissue was dissected. (c) The common carotid artery and external carotid artery were ligated. (d) The wound was disinfected and sutured

The rat model of the focal MCAO was established by the thread plug method. Before modelling, the threaded plug and surgical instruments were soaked in 75% medical alcohol for 12 HRS in advance, and then sterilised by high‐pressure steam. Then, the rats were anaesthetized with 10% chloral hydrate, and placed supine on the operating table with neck leaking. After iodophor disinfection, choosing the intersection of the line of the mandible and the journal axis, 0.5 cm at the right side of it, an incision of about 1–2 cm was made. The arterial sheath was found and separated, together with the vagus nerve, common carotid artery (CCA), internal carotid artery (ICA) and external carotid artery (ECA). ECA was located at the furcation of ICA and ECA, and CCA was located 1–1.5 cm from the furcation proximal to the heart. CCA was clamped with an arterial clamp. A small incision was made on the CCA with ophthalmic scissors at the lower end of the artery clip, and the thread was fixed, so that it could slowly enter ICA. It stopped when the head of the thread was about 18 mm from the cross of ICA and ECA, and the thread was fixed again. The tissue and skin were sutured after surgery, and all animals were intraperitoneally injected with penicillin (100 U/d) to prevent infection. The schematic diagram of MCAO model production is shown in Figure 1.

### 2.9 qRT‐PCR analysis

The expression of 10 hub genes in peripheral blood mononuclear cells (PBMCs) was verified by qRT‐PCR. First, total RNA was isolated from each sample by TRIZOL Reagent (Invitrogen, Carlsbad, USA). After that, reverse transcription was performed through TAKARA PrimeScript RT Master Mix (RR036A), and qRT‐PCR with SYBR Green Premix (RR420A). Finally, the relative quantification was identified by the 2^−Δ∆CT^ method. The qRT‐PCR primers used in this study were shown in Table 2 (*p* < 0.05 was considered statistically significant).

**TABLE 2:** The primers used in qRT‐PCR

Primers	Forward	Reverse
β‐actin	5′‐TGTCACCAACTGGGACGATA‐3′	5′‐GGGGTGTTGAAGGTCTCAAA‐3′
CXCL8	5′‐CCCCCATGGTTCAGAAGATTG‐3′	5′‐TTGTCAGAAGCCAGCGTTCAC‐3′
IL‐6	5′‐ACTTCCAGCCAGTTGCCTTCTTG‐3′	5′‐TGGTCTGTTGTGGGTGGTATCCTC‐3′
IL‐1B	5′‐AATCTCACAGCAGCATCTCGACAAG‐3′	5′‐TCCACGGGCAAGACATAGGTAGC‐3′
ICAM1	5′‐TGTCGGTGCTCAGGTATCCATCC‐3′	5′‐TTCGCAAGAGGAAGAGCAGTTCAC‐3′
CCL4	5′‐CCACTTCCTGCTGCTTCTCTTACAC‐3′	5′‐GCCAGTTTCCTGTCATTCCCTCAC‐3′
CXCL1	5′‐GCAGACAGTGGCAGGGATTCAC‐3′	5′‐TGAGTGTGGCTATGACTTCGGTTTG‐3′
CXCL2	5′‐TGAAGTTTGTCTCAACCCTGAAGCC‐3′	5′‐AGGTCAGTTAGCCTTGCCTTTGTTC‐3′
PTGS2	5′‐CACATTTGATTGACAGCCCACCAAC‐3′	5′‐AGTCATCAGCCACAGGAGGAAGG‐3′
IL‐1	5′‐AAAGCCTGTGTTGCTGAAGGAGATTC‐3′	5′‐CTCTGGGAAAGCTGCGGATGTG‐3′
CXCL20	5′‐GGACACAGCCCAAGGAGGAAATG‐3′	5′‐GGACAAGACCACTGGGACACAAATC‐3′

## 3 RESULTS

### 3.1 Flow chart of the present study

**FIGURE 2:**
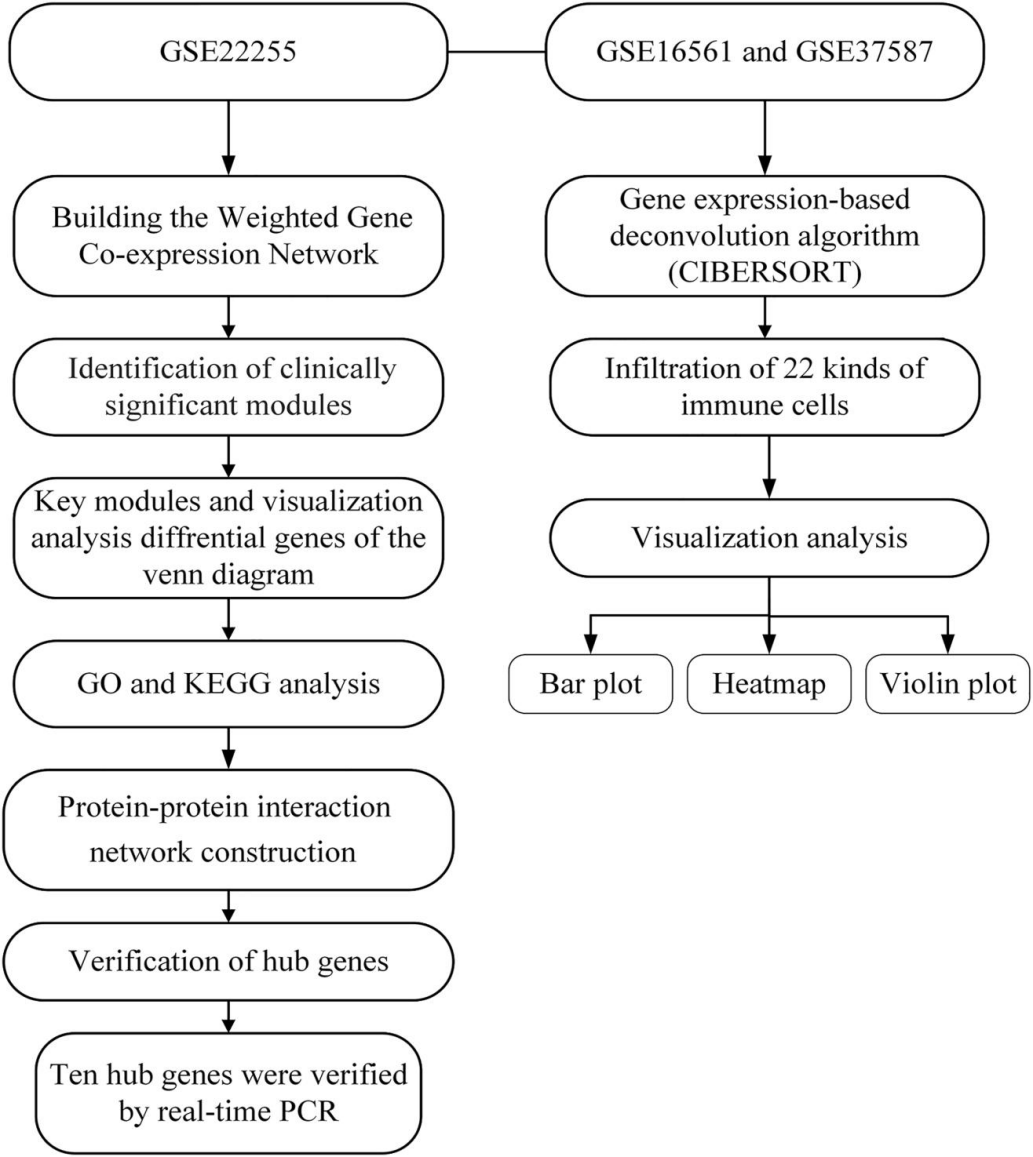
The steps of the current work

The gene expression profiles, GSE16561, GSE22255 and GSE37587, were downloaded from the GEO database. GSE22255 was used to construct WGCNA. Then, GSE16561 and GSE37587 were used for the related immune infiltration analysis of sex differences in IS. The specific process was shown in Figure 2.

### 3.2 Identification of differential genes

**FIGURE 3:**
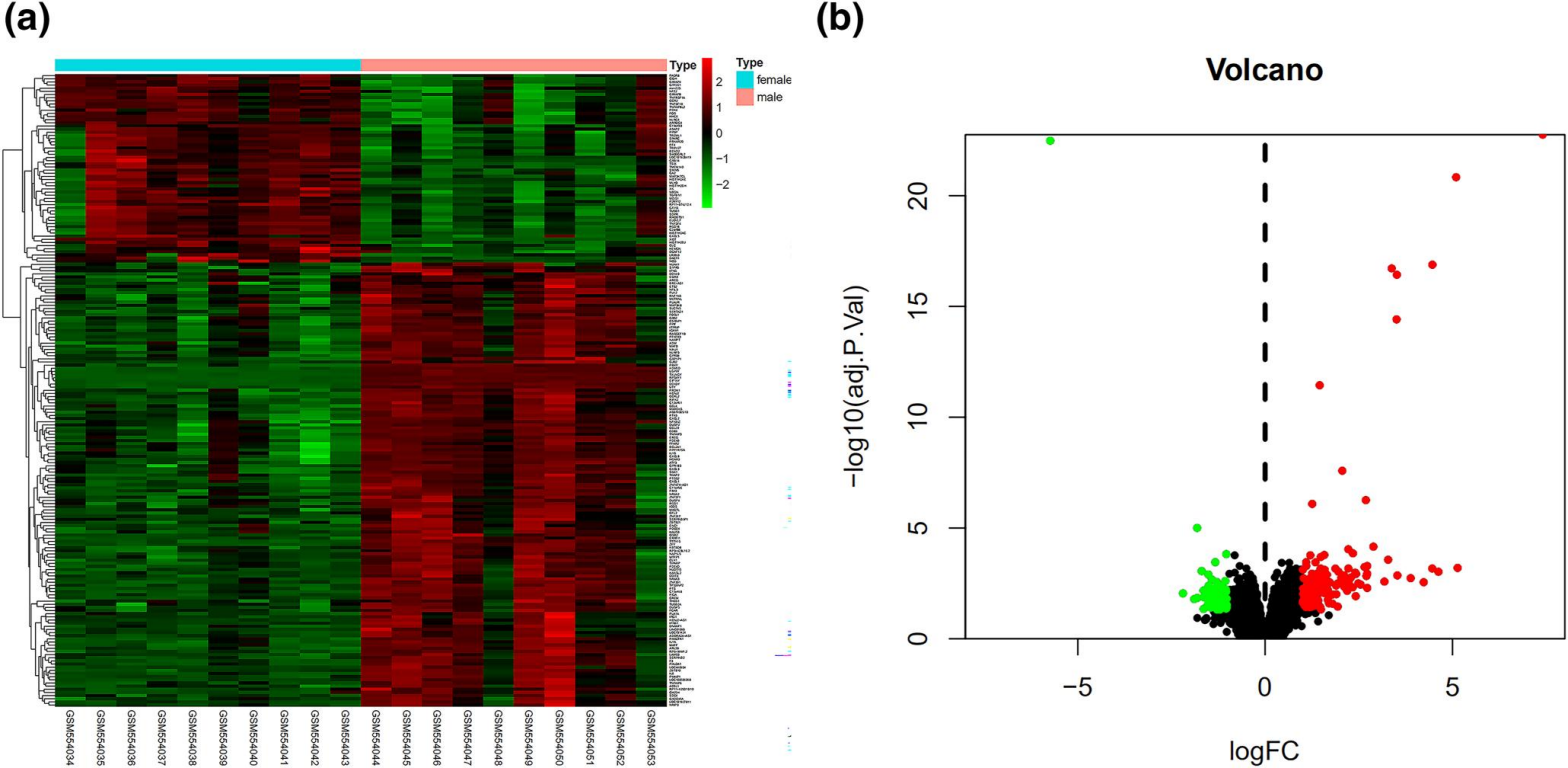
Differential expression analysis. (a) Differential genes of males and females with IS. (b) Volcano plot of DEGs. Up‐regulated and down‐regulated DEGs were shown as red and green, respectively

First, the GSE22255 data set was chosen and the peripheral blood data of 10 male and 10 female IS patients were selected from the GSE22255 data set for subsequent analysis. According to the selection criteria (|log2FC| ≥ 1, *p* < 0.05), GSE22255 data set were analysed with limma packet, 202 DEGs were identified, including 141 up‐regulated genes and 61 down‐regulated genes, then the selected DEGs were analysed with heat map and volcano plot, which could show the differential genes of male and female patients with IS (Figure 3).

### 3.3 Construction of weighted co‐expression network

**FIGURE 4:**
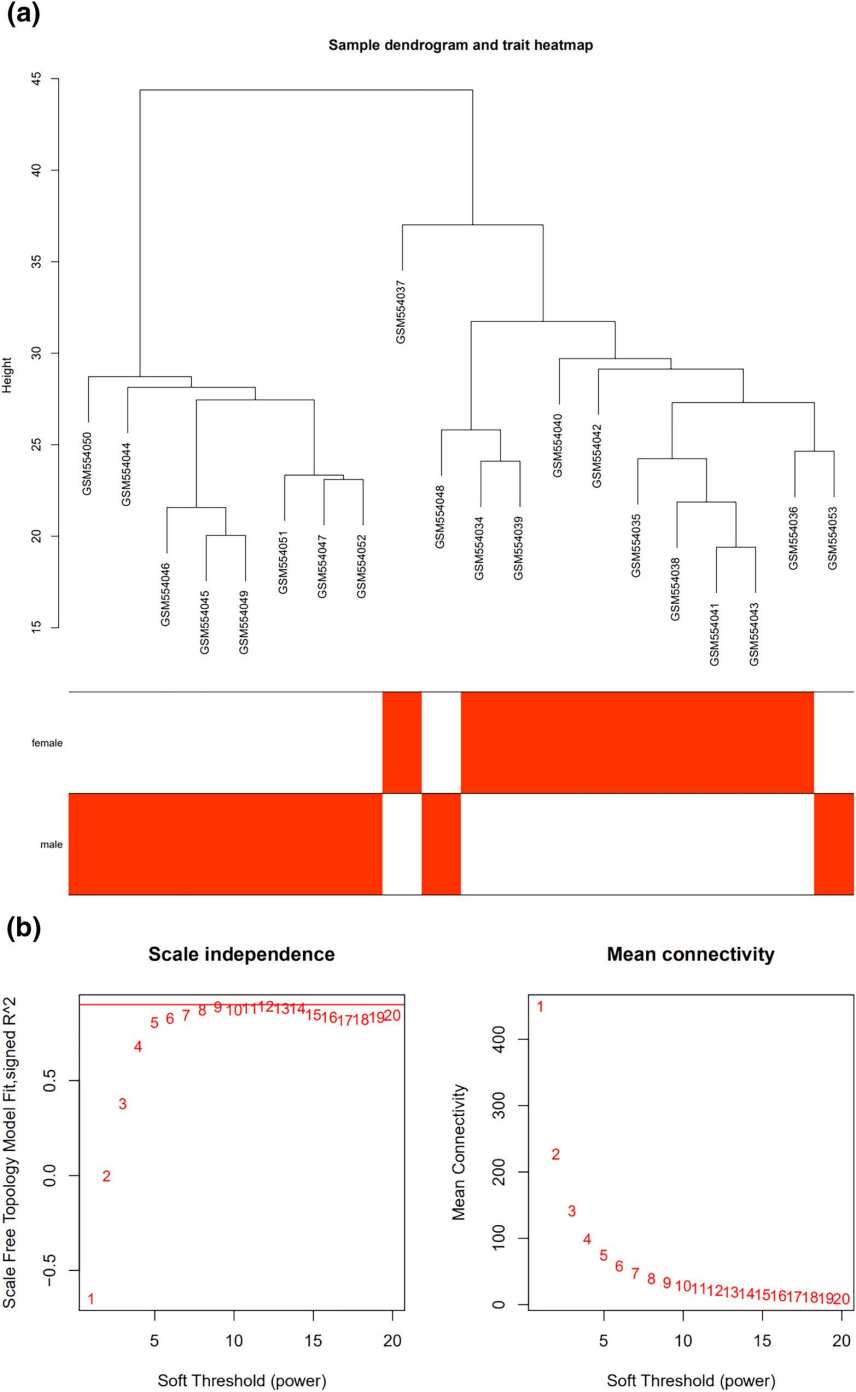
The selection optimal soft threshold power to construct gene co‐expression networks. (a) Cluster tree diagram of 20 samples. The cluster tree reflects the relationship between 20 samples. (b) Analysis of network topology for various soft thresholds. The *X*‐axis represents soft threshold power. The *Y*‐axis represents the scaling topology model fitting index

To identify the core genes responsible for sex differences in IS, we constructed WGCNA for the GSE22255 data set. First, the inclusion criteria samples were dealt with clustering analysis (Figure 4). Then, we determined the appropriate soft threshold, which was used to construct a weighted gene co‐expression network, and divided co‐expression modules through the dynamic cutting and module merging. We set the threshold of 0.25 to merge with five co‐expression modules received.

### 3.4 Identification of significant modules

**FIGURE 5:**
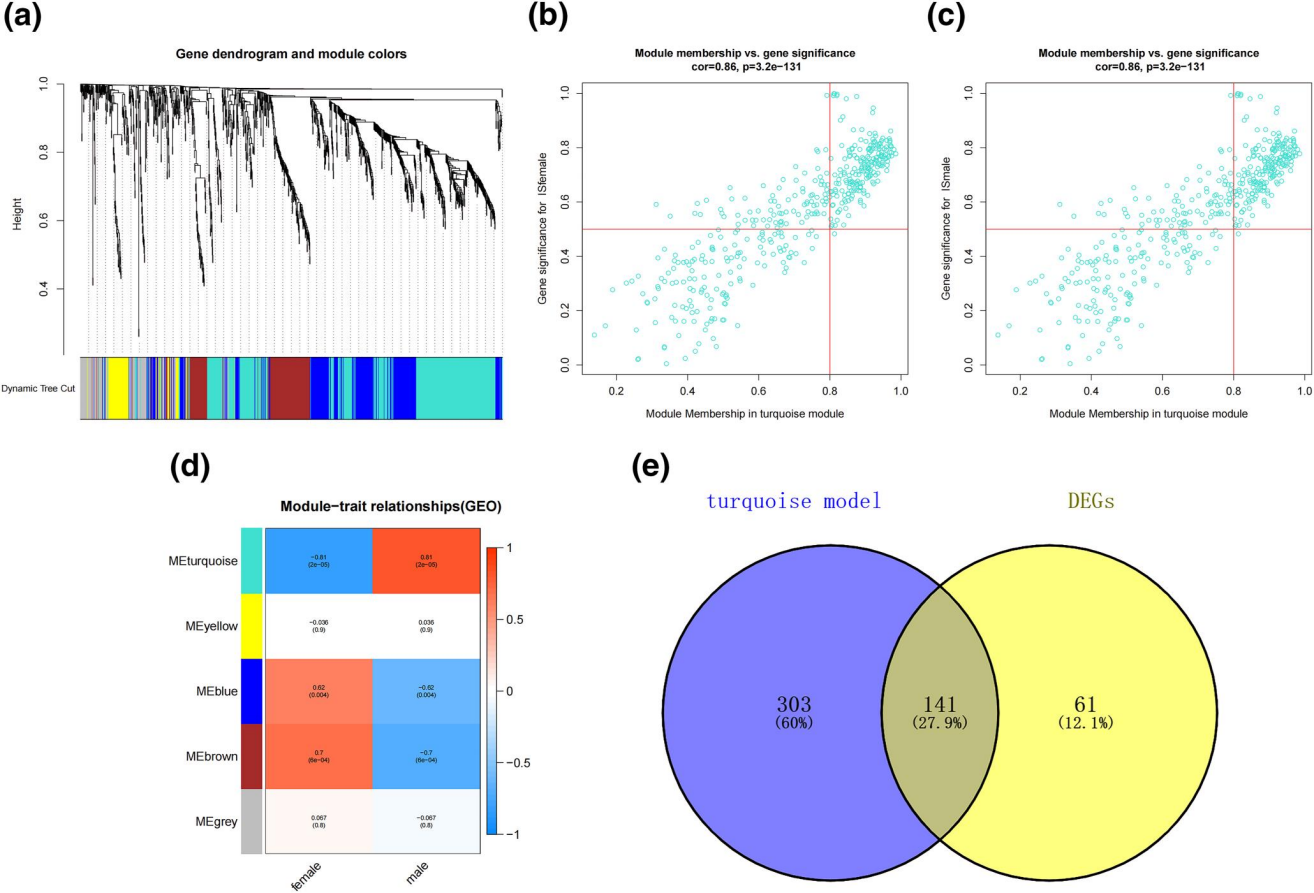
Identification of modules specifically associated with sex differences in IS. (a) Cluster tree diagram and module feature relation diagram are used to divide modules. (b) Heat map of the correlation between module characteristic genes and different stages. Each cell contains a specific associated exponent and *p*‐value. (c) Scatter diagram of female patients with IS in the turquoise module. (d) Scatter diagram of male patients with IS in the turquoise module. (e) Turquoise modules with differential gene Venn diagrams, a total of 141 common genes

The satisfactory IS samples were dealt with correlation analysis, and five gene expression modules were constructed finally, including turquoise module, yellow module, blue module, brown module and grey module (Figure 5a). Among them, the turquoise module had the highest correlation with sex differences in IS: *r* = 0.81 (Figure 5b). Therefore, genes of the turquoise module were selected for the subsequent analysis (Figure 5c and d). To identify the core genes of sex differences more precisely, we intersected the most clinically significant module (turquoise module) with the differential genes (Figure 5e).

### 3.5 Functional enrichment analyses of genes in key modules

**FIGURE 6:**
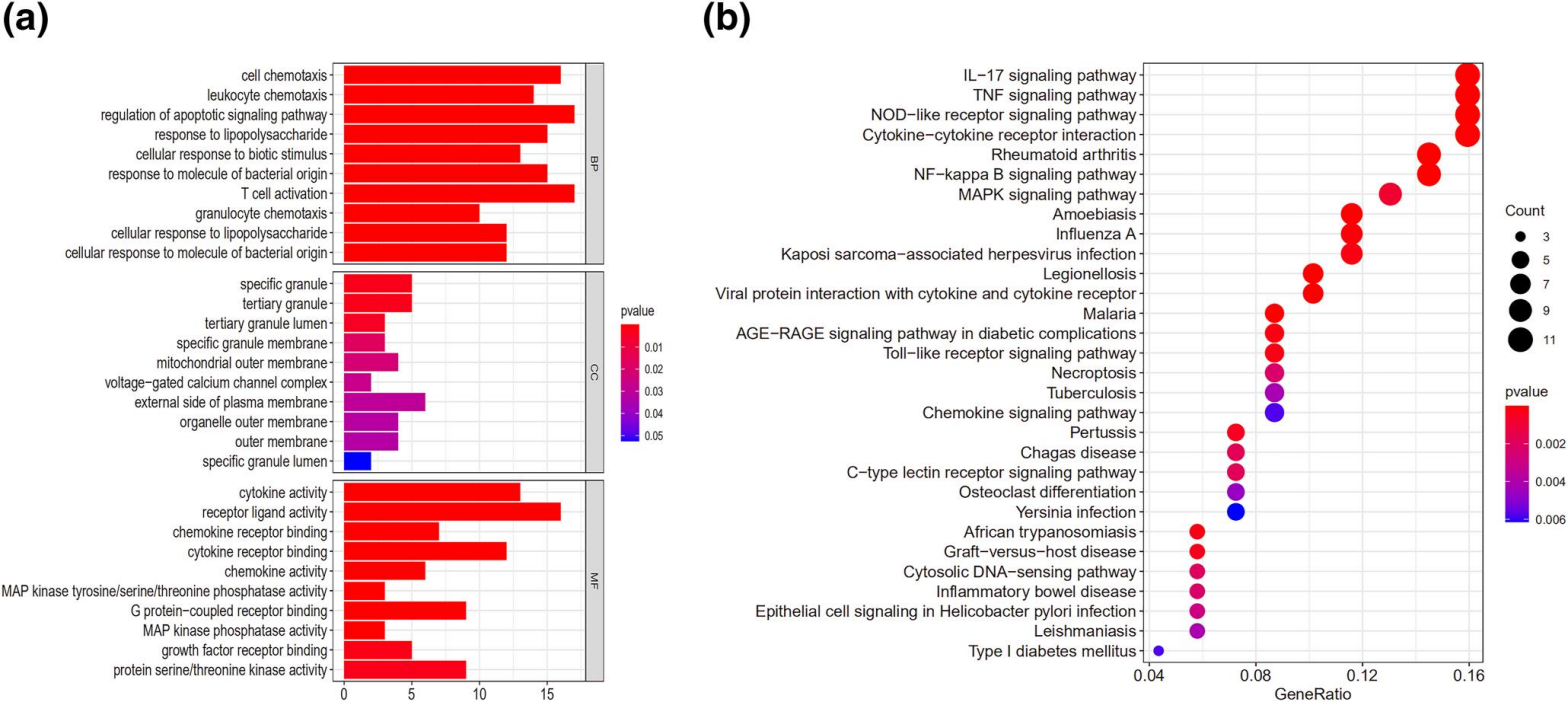
Functional enrichment analysis of the genes in the key modules. (a) GO enrichment analysis of sex differences in IS. (b) KEGG enriched the regulatory pathways of sex differential expressed genes in IS

The turquoise module contained 303 genes, and the number of differential ones between males and females was 202. According to the analysis, there were 141 common genes between the genes in turquoise module and DEGs. Therefore, in this study, 141 genes were analysed. As shown in GO results, the biological process (BP) of sex differences in ischaemic stroke was mainly manifested in T cell activation, regulation of apoptotic signalling pathway, cell chemotaxis and so on. Cellular constituent (CC) genes were mainly enriched in specific granule, tertiary granule and external side of plasma membrane etc, while molecular function (MF) was mainly in receptor‐ligand activity, cytokine activity, cytokine receptor binding and other aspects (Figure 6a). Subsequently, we performed KEGG analysis on the common genes and identified the main regulatory ones for sex differences in IS, and the results showed that the IL‐17 signalling pathway, TNF signalling pathway, and NOD‐like receptor signalling pathway were the main ones (Figure 6b).

### 3.6 Analysis of sex differences related to immune infiltration of IS

**FIGURE 7:**
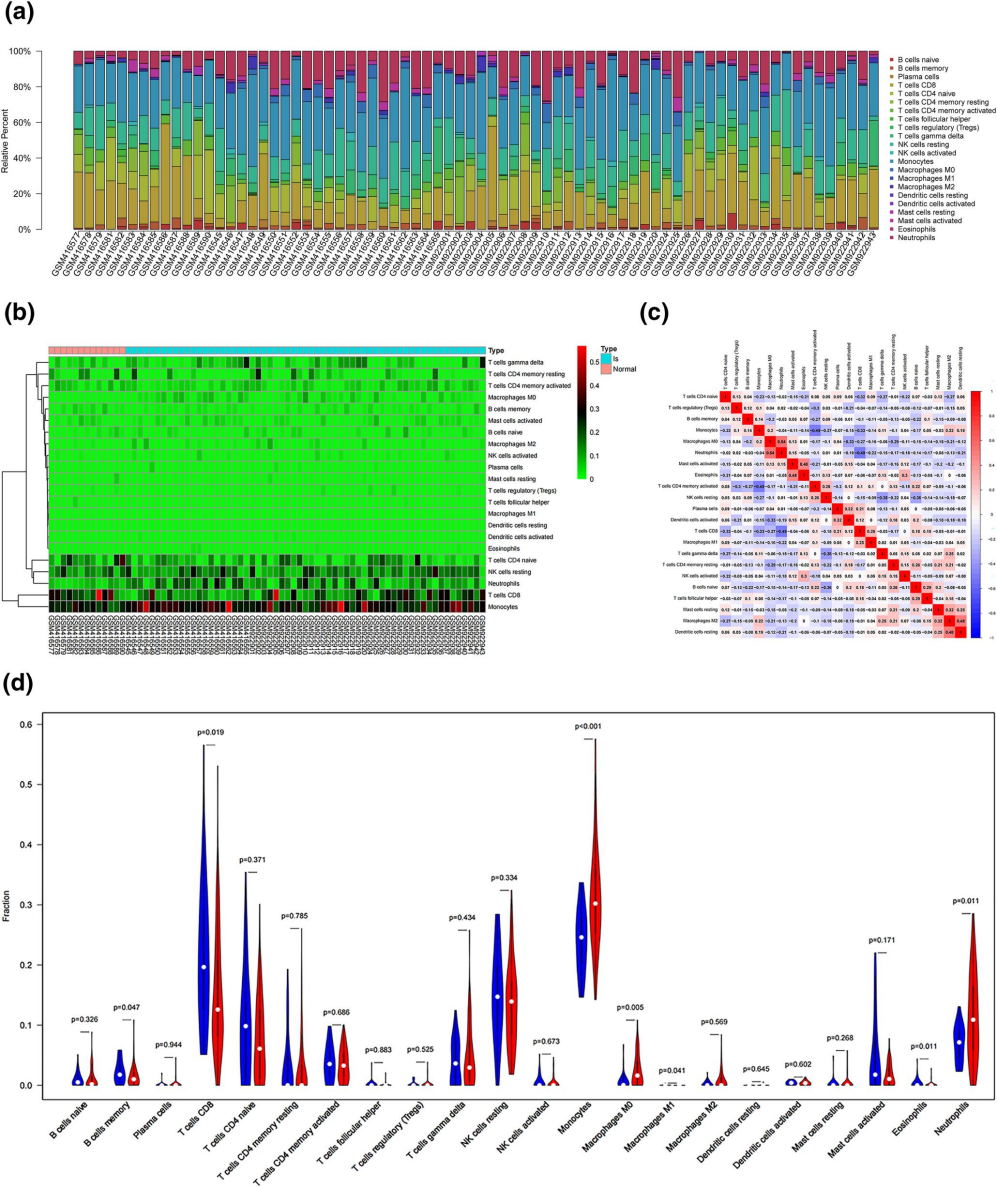
Immune infiltration situation in peripheral blood samples from females with IS and healthy ones. (a) Histogram of percentage distribution of 22 immune cell subtypes. (b) Hot map of percentage distribution of 22 immune cell subtypes. (c) Heat map of 22 immune cells in both samples. Positive and negative correlations were shown as red and blue, respectively. (d) Violin plot of differences in immune cell infiltration between females with IS and healthy ones. Blue, healthy females; Red, IS females

To further study the sex differences in IS, we studied the distribution of 22 immune cell subsets in male and female patients and compared them with healthy controls. As shown in Figure 7a and b, the two pictures, respectively, showed the distribution of various immune cells of female patients and female healthy controls. We observed that in females with IS, Monocytes, B cells memory, Neutrophils, T cells CD4 memory resting, T cells CD8, NK cells resting, T cells follicular helper, and T cells regulatory (Tregs) were the main immune infiltrating cells. In addition, as shown in Figure 7c, there was a correlation between different immune cells in female patients with IS and healthy ones. For example, the correlation between Neutrophils and Macrophages M0 was 0.54, T cells were negatively correlated with Monocytes, and the correlation was −0.49. In the samples of female patients and healthy ones, we found that, compared with the healthy, the proportion of T cells CD8 decreased, while Monocytes, Macrophages M0, Macrophages M, and Neutrophils increased (Figure 7d).

**FIGURE 8:**
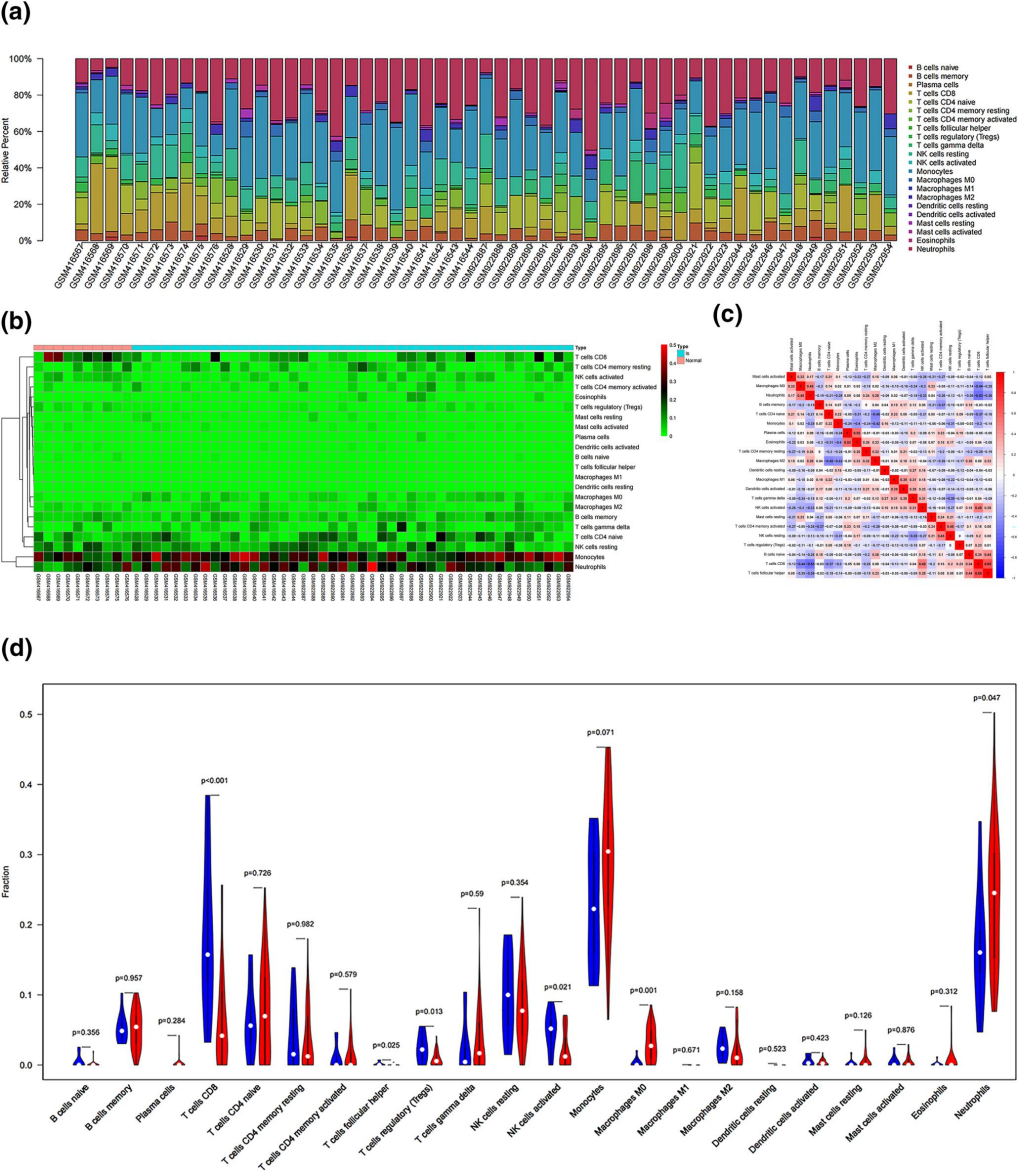
Immune infiltration situation in peripheral blood samples from males with IS and healthy ones. (a) Histogram of percentage distribution of 22 immune cell subtypes. (b) Hot map of percentage distribution of 22 immune cell subtypes. (c) Heat map of 22 immune cells in both samples. Positive and negative correlations were shown as red and blue, respectively. (d) Violin plot of differences in immune cell infiltration between males with IS and healthy ones. Blue, healthy males; Red, IS males

We then selected samples from male patients with IS for immune infiltration analysis. As shown in Figure 8a and b, between male patients and healthy ones, B cells naive, Neutrophils, B cells memory, Monocytes, NK cells resting and T cells CD8 were the main immune cells in the course of IS in males. In the correlation heat map, we found that the correlation between T cells follicular helper and T cells CD8 was 0.65, T cells CD8 were negatively correlated with Neutrophils, and the correlation was −0.53 (Figure 8c). In the violin plot, it could be found that, compared with healthy ones, T cells CD8 and T cells follicular helper of male patients was reduced, while Neutrophils and Macrophages M0 increased (Figure 8d).

### 3.7 PPI network analysis and candidate hub genes

**FIGURE 9:**
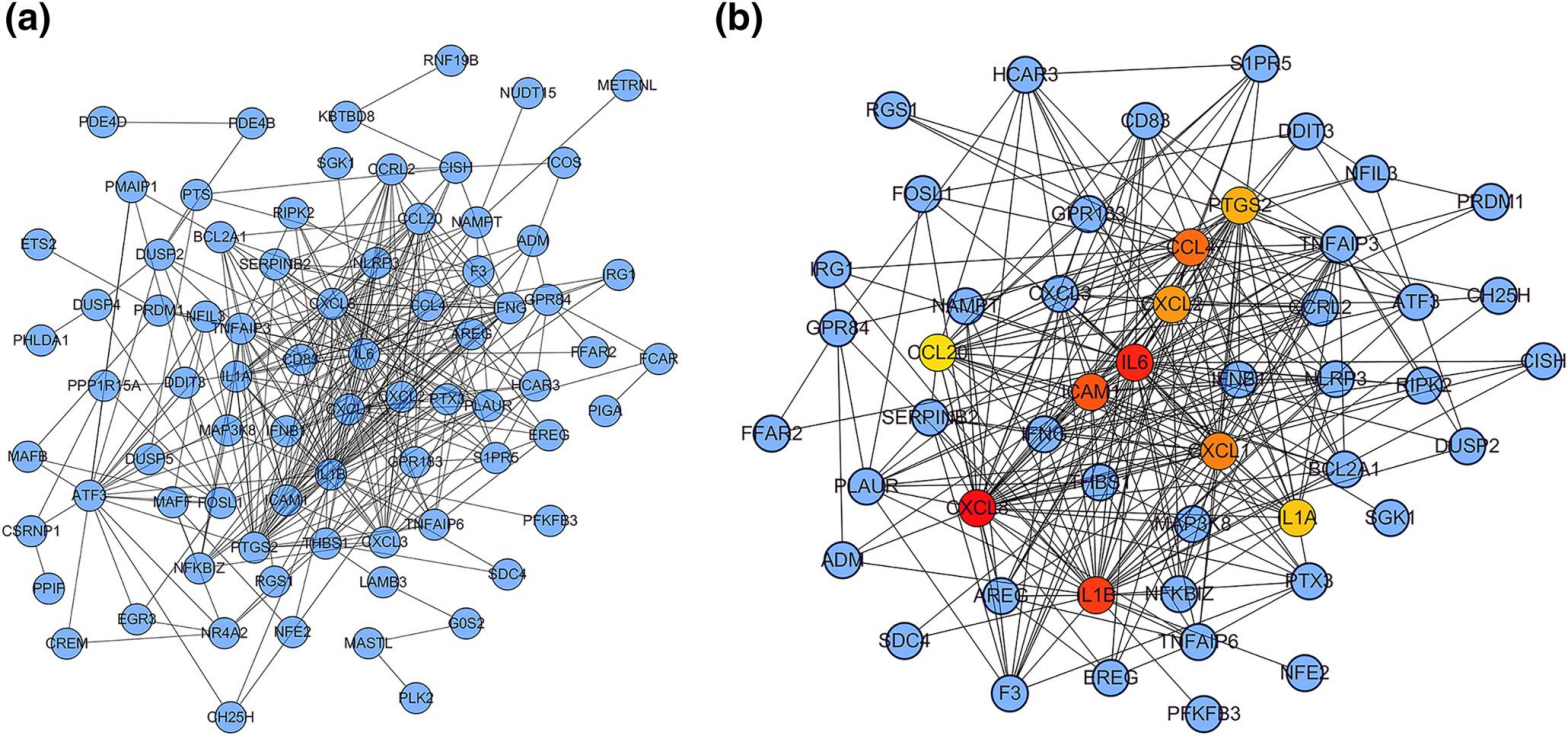
Construction of protein‐protein interaction networks. (a) Protein‐protein interaction (PPI) networks associated with sex differences in ischaemic stroke. (b) Core gene clusters in co‐expression networks, and 10 hub genes were screened out

To further explore sex differences in IS and look for potential biomarkers to distinguish them, we used the STRING database to construct PPI network for the most clinically significant module (turquoise module) and the intersection genes of differential ones (Figure 9a). On basis of the PPI network, we used the Cytohubba tool to identify hub genes according to their node degrees calculated (CXCL8, IL6, IL1B, ICAM1, CCL4, CXCL1, CXCL2, PTGS2, IL1A, and CCL20). The result of Cytohubba analysis is reported in Figure 9b.

### 3.8 Validation of the hub genes by qRT‐PCR

**FIGURE 10:**
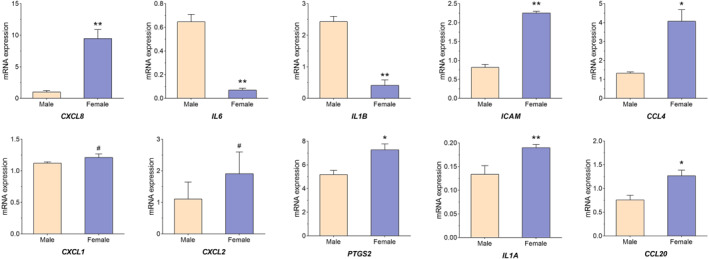
Validation of the hub genes by qRT‐PCR. Pale pink, male samples; blue, female samples. *means *p*‐value < 0.05, **means *p*‐value < 0.01, and #means no difference

To validate the results of WGCNA, the first 10 hub genes were selected (CXCL8, IL6, IL1B, ICAM1, CCL4, CXCL1, CXCL2, PTGS2, IL1A and CCL20) to be verified by qRT‐PCR. We first established the MCAO model simulating the pathological process of ischaemic stroke. The MCAO model in rodents has been accepted as the classical model to study IS in humans [15]. Afterwards, we collected PBMCs from MCAO model rats to measure the expression levels of 10 hub genes. As shown in Figure 10, the expressions of CXCL8, ICAM1, CCL4, PTGS2, IL1A, and CCL20 in female IS rats were significantly higher than those in male ones. In addition, compared with male IS rats, the expression of IL‐6 and IL1B in the female group significantly decreased, and there was no significant difference of CXCL1 and CXCL2 between the two groups.

## 4 DISCUSSION

Ischaemic stroke is a nervous system disease with high morbidity and mortality, which is also one of the major causes of lifelong disability in adults [16, 17]. Previous studies [18] have found that the aetiology, risk factors, and prognosis of the disease vary by different genders. For instance, females have a longer life expectancy, and IS is a common complication during pregnancy and puerperium, especially for females in high‐risk groups, with a higher incidence of the disease. Hence, females with IS are also less likely to recover. Males, on the other hand, have a higher incidence of stroke and are more likely to recover at a given age. In terms of treatment, there has been evidence that thrombolytic therapy is more effective for females than for males, and males have better neurological scores than females in the non‐thrombolytic administration group [19]. Interestingly, this conclusion is supported by data from the intravascular intervention trials. In addition, an increasing number of scholars come to realize that, after stroke, some inflammatory pathways may play a neuroprotective role and also contribute to tissue repair in the future [20]. Actually, in particular, the interaction of various immune cell types, and the dynamic relationship between immune cells and inflammatory factors, can help develop new immunotherapeutic means. Therefore, it is of great significance to explore the sex differences in IS, and to study the distribution pattern of peripheral blood immune cell subtypes in males and females patients for the diagnosis and treatment of this disease [21].

In this study, WGCNA and CIBERSORT algorithms were used to explore the core genes of sex differences in IS. First, we screened the downloaded data and identified 202 gene‐specific genes. Subsequently, we constructed the weighted gene network, made the intersection between the most significant clinical module (turquoise module) and differential genes, and all the intersected genes were analysed by GO and KEGG. Next, we conducted immune infiltration analysis on the samples, and found that compared with healthy females, the proportion of T cells CD8 decreased, while Monocytes, Macrophages M0, Macrophages M, and Neutrophils increased. Compared with healthy males, T cells CD8 and T cells follicular helper of male patients were reduced, while Neutrophils and Macrophages M0 increased. We also constructed a protein‐protein interaction network and identified 10 hub genes (CXCL8, IL6, IL1B, ICAM1, CCL4, CXCL1, CXCL2, PTGS2, IL1A, CCL20). Finally, we measured the expression of 10 hub genes to validate our results with qRT‐PCR method, and the qRT‐PCR verification results showed that our analysis was reliable.

Inflammation plays an important role in the occurrence and development of IS [22, 23]. Interleukin (ILS) is an effective regulator of the inflammatory process. Among the above 10 core genes, IL‐6, IL‐1B and IL1A belong to the family of interleukin cytokines. Studies have found [24] that interleukin 6 (IL‐6) is a multifunctional inflammatory cytokine with pro‐inflammatory and anti‐inflammatory properties. Elevated serum IL‐6 is associated with a high risk of cerebrovascular diseases. IL‐6 released after stroke can aggravate cerebrovascular damage by activating NMDI‐Rs and up‐regulating the enhancement of ET‐1 and JNK. Moreover, IL‐6, a gene promoter, is the main predictor for stroke recurrence in young adults with moderate internal carotid artery stenosis [25, 26].

The inflammatory cytokine interleukin (IL) −1 plays a key role in regulating the immune response after IS and is a potential target for stroke therapy. IL‐1 includes two agonists, IL1A (IL‐1α) and IL1B (IL‐1β) [27]. L1B (IL‐1β) are the main medium of central and peripheral inflammation after stroke. Through the effects on neurons, glia, and vasculature, it mediates ischaemic, traumatic and excitatory toxic brain injuries [28]. Many clinical studies are committed to improving the level of IL‐1β in animal models, so as to lower the promotion of cardiovascular function [29, 30]. In addition, IL‐1β may be a useful biomarker for early detection of relapse after the first epileptic seizure in patients with ischaemic stroke [31]. Previous studies have shown that IL‐1α and IL‐1β play different roles in various inflammatory modes. In ischaemic stroke, the expression of IL‐1α precedes that of IL‐1β. In the acute phase, IL‐1α is highly expressed in focal neurons and microglia [32], and the administration of IL‐1α has the function of neural protection and repair after IS [33]. Our results indicated that IL‐6, IL‐1B, and IL1A may play a significant role in the sex differences in IS.

In the development of IS, two chemokines, CC and CXC, are involved in the regulation of key links in this disease, which can regulate atherosclerotic plaque vulnerability and the inflammatory response of cerebral infarction. Besides, CXC chemokine can also regulate the homing of stem cells, which is conducive to tissue repair, and maybe a potential therapeutic target for stroke [34]. CXCL8 may affect the development of IS through regulating PI3K/Akt/NF‐κB signalling pathway. CXCL8 silencing can significantly reduce the infarct area and improve neurological function in mice [35]. Retinoic acid Am80 promotes the recovery of nerve function after cerebral haemorrhage in mice by inhibiting the up‐regulation of CXCL2 [36]. CXCL1 is a neutrophil attractant chemokine. Studies [37] have found that progesterone can inhibit the secretion of MCP‐1 and CXCL1 by endothelial cells after IS, and reduce the infiltration of white blood cells, thus promoting the recovery of cerebrovascular vessels, and CCL4 may be an important biomarker for the prognosis of patients with IS [38]. Previous studies have found that CCL20 is closely related to neurodegenerative diseases after traumatic brain injury [39]. However, the dysfunction of CCL20 in ischaemic stroke has not been reported before. In our study, the expressions of CCL20 in female IS rats were significantly higher than those in male ones.

ICAM1, a member of the immunoglobulin superfamily (IGSF) of adhesion molecules, is an important adhesion molecule that mediates adhesion response. Related studies [40] have found that ICAM‐1 increases in the early exercise after stroke, which may exacerbate the damage of brain tissue. Also, ICAM‐1 has a close relationship between hypertensive state and stroke risks of females [41]. Furthermore, genetic variation of PTGS2 is associated with carotid plaque vulnerability in patients with IS, and specific knockdown of PTGS2 can inhibit the NF‐κB signalling pathway in mice and has a protective effect on the mouse model of IS [42, 43]. However, the association of ICAM1 and PTGS2 expression with sex differences in ischaemic stroke has not been previously reported. In the present study, it was found that the expressions of ICAM and PTGS2 in female IS were significantly higher than those in male ones.

Previous studies have shown that the neuroinflammatory response to IS is primarily characterised by invasion of the damaged blood‐brain barrier by peripheral immune cells, followed by activation of innate immune cells, which together produce many inflammatory mediators, namely cytokine, growth factors and chemokines [44]. For example, T cells CD8 promote the inflammatory response of IS and aggravate brain damage and neurological impairment [45]. Neutrophils is the main cell in focus within 3 days after IS lesions. The increase of neutrophils was positively correlated with infarct volume and function defects. Monocytes/macrophages play a dual role in IS, mainly depend on the microenvironment of the IS and time window [46], which is also similar to our immunity infiltration results (Figures 7 and 8). In addition, the 10 Hub genes identified in this study were mainly cytokines and chemokines. Therefore, we speculate that these genes are closely related to T cells CD8, Monocytes and Macrophages. In male and female patients, targeted regulation of T cells CD8, Monocytes and Macrophages homoeostasis may be an important method to treat IS.

In conclusion, we explored the differences between male and female patients with IS from two aspects, such as WGCNA and immune infiltration. Among 10 hub genes, the role of seven genes (CXCL8, IL6, IL1B, CCL4, CXCL1, CXCL2 and IL1A) in IS have been reported. Besides, CCL20, ICAM1 and PTGS2 had been less studied in IS, and these might be potential biomarkers for sex differences in IS. In addition, this study has several limitations. For example, the mechanisms of several central genes for sex differences in IS are unclear. In addition, the validity of different immune cells we identified in sex differences needs to be evaluated.

## 5 CONCLUSIONS

In this study, we identified five gene expression modules and 10 central genes. Among them, CCL20, ICAM1 and PTGS2 had been less studied in IS, and they might be important biomarkers for sex differences in IS. Besides, the hub genes were further verified by rat experiments. In addition, we found that there were sex differences in the distribution of immune cells in patients with IS. This finding might provide new ideas for the immunotherapy of IS.

## CONFLICT OF INTEREST

The authors declare that they have no conflicts of interest.

## Data Availability

All data can be obtained from corresponding author Qianqian Liu or the first author Haipeng Xu.
